# Overexpressed *BSR1*-Mediated Enhancement of Disease Resistance Depends on the MAMP-Recognition System

**DOI:** 10.3390/ijms21155397

**Published:** 2020-07-29

**Authors:** Yasukazu Kanda, Yoko Nishizawa, Takashi Kamakura, Masaki Mori

**Affiliations:** 1 Institute of Agrobiological Sciences, NARO (NIAS), Tsukuba 305-8602, Japan; kanday@affrc.go.jp (Y.K.); ynishi@affrc.go.jp (Y.N.); 2 Department of Applied Biological Science, Graduate School of Science and Technology, Tokyo University of Science, Noda 278-8510, Japan; kamakura@rs.noda.tus.ac.jp

**Keywords:** disease resistance, microbe-associated molecular pattern (MAMP), *Pyricularia oryzae* (formerly *Magnaporthe oryzae*), *Oryza sativa* (rice), receptor-like cytoplasmic kinase (RLCK)

## Abstract

Plant plasma membrane-localized receptors recognize microbe-associated molecular patterns (MAMPs) and activate immune responses via various signaling pathways. Receptor-like cytoplasmic kinases (RLCKs) are considered key signaling factors in plant immunity. BROAD-SPECTRUM RESISTANCE 1 (BSR1), a rice RLCK, plays a significant role in disease resistance. Overexpression of *BSR1* confers strong resistance against fungal and bacterial pathogens. Our recent study revealed that MAMP-triggered immune responses are mediated by BSR1 in wild-type rice and are hyperactivated in *BSR1*-overexpressing rice. It was suggested that hyperactivated immune responses were responsible for the enhancement of broad-spectrum disease resistance; however, this remained to be experimentally validated. In this study, we verified the above hypothesis by disrupting the MAMP-recognition system in BSR1-overexpressing rice. To this end, we knocked out *OsCERK1*, which encodes a well-characterized MAMP-receptor-like protein kinase. In the background of BSR1 overaccumulation, the knockout of *OsCERK1* nearly abolished the enhancement of blast resistance. This finding indicates that overexpressed *BSR1*-mediated enhancement of disease resistance depends on the MAMP-triggered immune system, corroborating our previously suggested model.

## 1. Introduction

Plants can detect approaching microbes by recognizing microbe-associated molecular patterns (MAMPs), and in response activate various immune responses, which are referred to as pattern-triggered immunity (PTI) [1]. PTI acts as a barrier to a broad spectrum of indigenous microbial species. Numerous factors are involved in PTI. Receptor-like kinases (RLKs) and receptor-like proteins on the plasma membrane form pattern-recognition receptor complexes to perceive MAMPs and activate intracellular signaling pathways [2]. Activated signaling factors regulate downstream responses, such as the production of reactive oxygen species (ROS) and the transcriptional activation of defense-related genes, followed by signal transduction via plant hormones and phytoalexin biosynthesis [1,3,4]. Virulent microbes (i.e., pathogens) have evolved various means to avoid triggering PTI in host plants, including the secretion of effectors and structural variations of MAMPs [5].

Despite the vast improvements in our knowledge of plant immunity and plant–pathogen interactions, our understanding of genes that confer broad-spectrum resistance against pathogens is currently limited. *BROAD-SPECTRUM RESISTANCE 1* (*BSR1*; OsRLCK278), which encodes a rice receptor-like cytoplasmic kinase (RLCK), is a broad-spectrum disease resistance gene. Overexpression of *BSR1* confers strong resistance against at least four pathogens in rice, i.e., *Pyricularia oryzae*, which causes rice blast, *Cochliobolus miyabeanus*, which causes brown spot disease, *Xanthomonas oryzae* pv. *oryzae*, which causes bacterial leaf blight, and *Burkholderia glumae*, which causes bacterial seedling rot [6,7]. RLCKs are characterized as RLK-homologous cytosolic protein kinases [8,9]. Accumulating evidence indicates that RLCKs act as phosphorylation signaling factors that are crucial for the activation of PTI [10]. Knockout of *BSR1* in rice strongly suppresses immune responses triggered by MAMPs, such as chitin, peptidoglycan, and lipopolysaccharide (LPS) [11,12]. The BSR1 protein possesses protein kinase activity [13]; this indicates that, in wild-type (WT) rice, it may mediate the intracellular phosphorylation signaling downstream of pattern recognition receptors. In *BSR1*-overexpressing rice, MAMP-triggered immune responses are hyperactivated, resulting in enhanced disease resistance [12]. The broad-spectrum disease resistance induced by *BSR1* overexpression has been attributed to the hyperactivation of PTI; however, this remained to be experimentally validated.

Here, we knocked out *OsCERK1*, which encodes a plasma membrane-localized RLK, in *BSR1*-overexpressing rice to determine whether or not the enhancement of resistance depends on the MAMP-recognition system. OsCERK1 contributes to the recognition of various MAMPs, such as peptidoglycan, LPS, and chitin [14,15,16,17]. It coassembles with several receptor-like proteins into receptor complexes that recognize MAMPs [14,15]. Such complexes are considered to activate immune responses via a signaling pathway involving BSR1 [11].

## 2. Results

**Figure 1:**
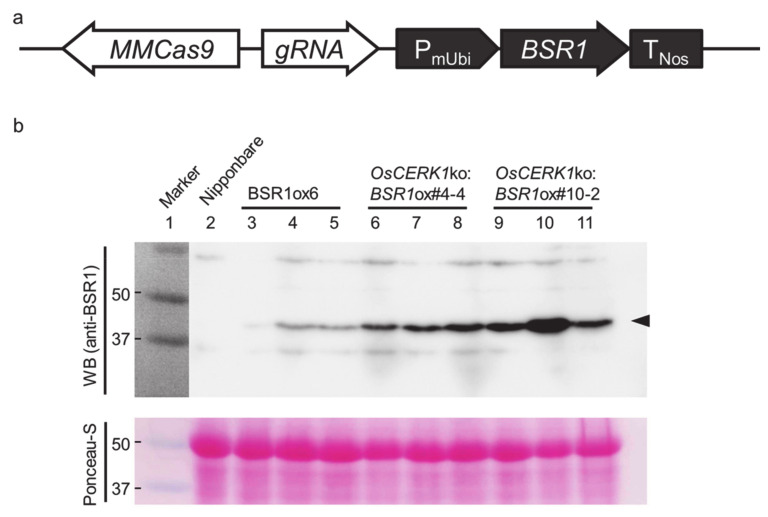
Generation of transgenic rice lines overexpressing *BSR1* and lacking *OsCERK1*. (**a**) Schematic representation of the construct for simultaneous *BSR1* overexpression and *OsCERK1* knockout. *BSR1* under the control of the maize *Ubiquitin* promoter was inserted into the T-DNA region of the pZH_MMCas9 CRISPR/Cas9 vector. A separate vector was prepared for each of target sites #4 and #10. Agrobacterium-mediated introduction of the vectors into rice resulted in transformant #4 and #10 lines, respectively. P_mUbi_, maize *Ubiquitin* promoter; T_NOS_, *NOS* terminator; gRNA, guide RNA. (**b**) Western blot analysis using anti-BSR1 antibody showing the levels of accumulated BSR1 protein in WT (Nipponbare), *BSR1*-overexpressing (*BSR1*ox6), and *BSR1*-overexpressing and *OsCERK1*-knockout (*OsCERK1*ko:*BSR1*ox#4-4 and #10-2) plants. Three T1 plants were used for each transgenic line. WB (anti-BSR1), western blot analysis using anti-BSR1 antibody; Ponceau-S, Ponceau-S staining before antibody staining; black arrowhead, BSR1 (44.7 kDa).

First, we attempted to knock out *OsCERK1* in a *BSR1*-overexpressing line using the CRISPR/Cas9 system [18]. Two independent CRISPR/Cas9 system-mediated cleavage sequences (target sites #4 and #10; Table 1) were designed in *OsCERK1* exon 1 (Table 1). Biallelic frameshift mutations in the sequences abolish almost all domains of OsCERK1, such as protein kinase domain whose activity is necessary for signaling function [16,17]. However, transgenic plants could not be regenerated for any of the two target sites. *OsCERK1* is not a lethal gene [16], indicating that *BSR1* overexpression is responsible for the failure to transform. It was suggested that *BSR1*-overexpressing rice might be resistant to Agrobacterium infection, as it is resistant to the bacterial pathogens *X. oryzae* pv. *oryzae* and *Burkholderia glumae* [7]. To avoid *BSR1* overexpression interfering with Agrobacterium infection, we next constructed a modified binary vector. It had a T-DNA region containing a *Cas9*-, *gRNA*-, and *BSR1*-constitutive expression cassette (Figure 1a) to concomitantly introduce *BSR1* and CRISPR/Cas9 components into WT rice. Modified binary vectors targeted to sites #4 and #10 were used for Agrobacterium-mediated rapid transformation. For both target sites, the cleavage site in each regenerated plant was sequenced, and insertion-deletion mutations were successfully detected (Table 1). Transgenic plants containing biallelic mutations which were expected to disrupt *OsCERK1* were selected for subsequent confirmation. CRISPR/Cas9-mediated insertion mutation in transgenic plant #4-4 generated a stop codon, and the plant was expected to express only N-terminal 41 amino acid residues of OsCERK1. Transgenic plant #10-2 was estimated to express N-terminal 33 amino acid residues followed by nonfunctional peptide generated by frameshift (Figure S1). The mutations were detected in *OsCERK1* transcripts from #4-4 and #10-2 lines by cDNA sequencing (Figure S2). Meanwhile, a *BSR1* overexpression vector pRiceFOX:*BSR1* was also introduced into WT rice. Three T1 plants for each transgenic line were subjected to western blot analysis using an anti-BSR1 antibody, which indicated that BSR1 was overaccumulated in the transgenic lines (Figure 1b). Transgenic lines #4-4 and #10-2 (designated as *OsCERK1*ko:*BSR1*ox#4-4 and #10-2, respectively) were used for further analyses, as they accumulated more BSR1 protein than *BSR1*ox6, a simple *BSR1*-overexpressing line.

**Figure 2:**
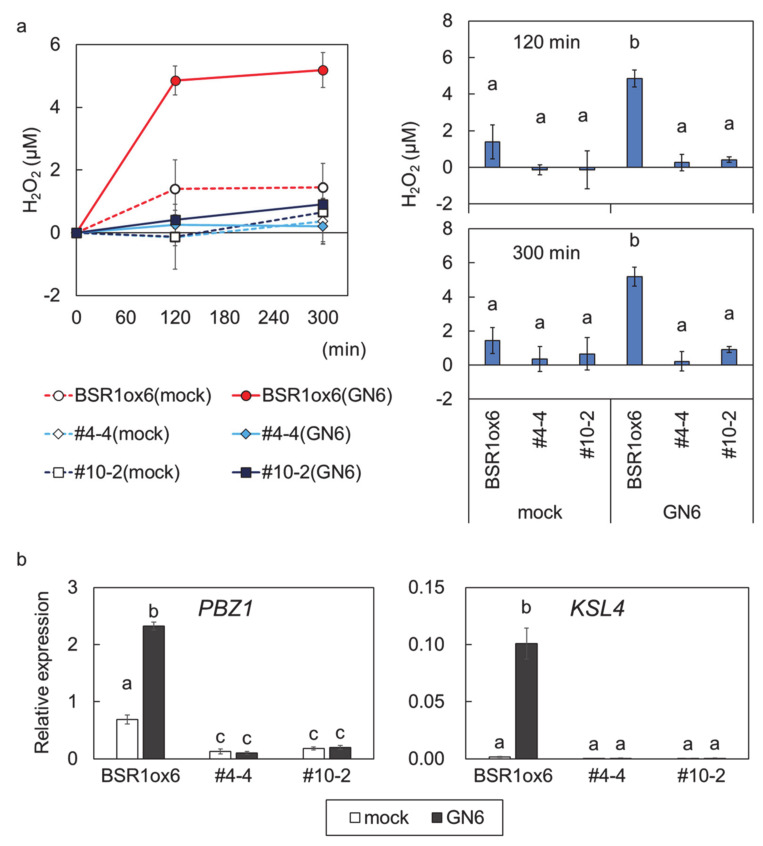
Knockout of *OsCERK1* completely abolishes chitin-triggered defense responses in *BSR1*-overexpressing leaf strips. (**a**) Chitin-triggered H_2_O_2_ production. H_2_O_2_ concentrations were measured by luminol-dependent chemiluminescence assay before treatment and at 120 min and 300 min after treatment. Left panel, time course of H_2_O_2_ concentration; right panel, data, with different letters indicating significant differences. (**b**) Transcript levels of defense-related genes in chitin-treated leaf strips. *PBZ1* and *KSL4* transcript levels at 5 h posttreatment were assessed by RT-qPCR. Values are presented as the means ± standard deviations of three biological replicates. Experiments were conducted twice, with similar results. Different letters indicate significant differences (Tukey’s test; *p* < 0.05). Mock, treatment with water; GN6, treatment with 100 nM chitin hexamer (*N*-acetylchitohexaose).

In rice, the recognition of chitin and subsequent activation of immune responses completely depends on OsCERK1 [14,16]. To ascertain whether the CRISPR/Cas9 system-mediated mutations disrupted *OsCERK1*, the chitin-responsivity of *OsCERK1*ko:*BSR1*ox lines was compared with that of the *BSR1*ox6 line. Leaf strips from leaf blades of all lines were treated with chitin as an elicitor. H_2_O_2_, a kind of ROS molecule produced during the immune response, was quantified after chitin treatment. Leaf strips from *BSR1*-overexpressing plants produced detectable amounts of ROS in response to chitin treatment, whereas those from *OsCERK1*ko:*BSR1*ox#4-4 and #10-2 plants displayed no significant chitin responsivity (Figure 2a). Transcriptional activation of defense-related genes was assessed by quantitative reverse-transcription (RT-q)PCR. *PROBENAZOLE-INDUCIBLE PROTEIN 1* (*PBZ1*) and *KAURENE SYNTHASE-LIKE 4* (*KSL4*), the expression of which is upregulated in response to chitin [12], were analyzed. Chitin treatment remarkably increased *PBZ1* and *KSL4* transcript levels in *BSR1*ox6 leaf strips, but not in *OsCERK1*ko:*BSR1*ox#4-4 and #10-2 leaf strips (Figure 2b). Similarly, the knockout of *OsCERK1* suppressed H_2_O_2_ production induced by peptidoglycan, a bacterial MAMP, in leaf strips under *BSR1*-overexpressing background (Figure S3). These results showed that the *OsCERK1*ko:*BSR1*ox#4-4 and #10-2 lines lacked responsivity to chitin, indicating that, as expected, the function of OsCERK1 was completely abolished by the insertion mutations.

**Figure 3:**
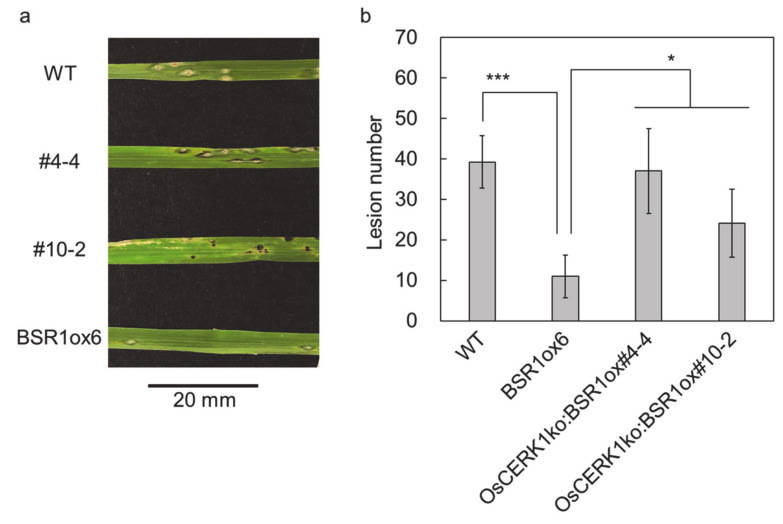
Knockout of *OsCERK1* impairs *BSR1* overexpression-mediated enhancement of rice blast resistance. (**a**) A photograph of the 4th leaf blades. (**b**) The number of compatible lesions on the 4th leaf blade counted on day 6 after inoculation. *Pyricularia oryzae* was spray-inoculated onto plants at the 3.5–4.0 leaf stage. Values are presented as the means ± standard deviations (*n* = 8, 8, 8, and 10 for WT, *BSR1*ox6, *OsCERK1*ko:*BSR1*ox#4-4, and *OsCERK1*ko:*BSR1*ox#10-2, respectively). Experiments were conducted twice, with similar results. * *p* < 0.05, Dunnett’s test for *OsCERK1*ko:*BSR1*ox#4-4 and *OsCERK1*ko:*BSR1*ox#10-2 vs. *BSR1*ox6; *** *p* < 0.001, *t*-test, WT vs. *BSR1*ox6. WT, wild-type; ox, overexpression; ko, knockout.

Resistance to rice blast was assessed in WT, *BSR1*ox6, and the two *OsCERK1*ko:*BSR1*ox lines. Plants were spray-inoculated with conidia of *Pyricularia oryzae*. When comparing the *BSR1*ox6 line with the WT line, *BSR1* overexpression was found to strongly suppress lesion formation (Figure 3), which is in line with findings in a previous report [5]. This indicates that BSR1 overaccumulation at the level observed in *BSR1*ox6 plants (Figure 1b) is sufficient to confer disease resistance. On the other hand, plants of the two independent *OsCERK1*ko:*BSR1*ox lines were significantly more susceptible than those of the *BSR1*ox6 line, despite the higher levels of accumulated BSR1 protein (Figure 1b and Figure 3). Together with previous reports that loss-of-function of OsCERK1 had no effect on the number of blast lesions formed in WT plants [16,19], this result indicated that knockout of *OsCERK1* impaired the enhancement of disease resistance by overaccumulation of BSR1.

## 3. Discussion

Until now, it was unclear whether OsCERK1 contributes to resistance against rice pathogens. Our data demonstrated the importance of OsCERK1 in PTI. In a previous study, knockout of *OsCERK1* completely abolished chitin responsivity in cultivated cells, but had no significant effect on lesion formation by a compatible rice blast fungal strain in leaf blades [16]. In a study using other compatible strain, the number of lesions formed was unaffected by knockdown of *OsCERK1*, although lesion size was increased [19]. In the current study, knockout of *OsCERK1* clearly suppressed the rice blast resistance of *BSR1*-overexpressing plants. Although in the background of *BSR1*-overexperssion, these data suggest, for the first time, that OsCERK1 contributes to the suppression of lesion formation by blast fungus in natural conditions (i.e., on leaf blades), and support the hypothesis that it recognizes MAMPs during blast infection, which is in line with previous observations in cultivated cells.

Overexpression of *BSR1* enhances disease resistance [6,7]. In a previously suggested model, the underlying mechanism was as follows: intracellular signaling following MAMP-recognition would be amplified via overaccumulation of BSR1, resulting in enhanced broad-spectrum immunity [12]. The simultaneous overexpression and knockout experiment in our study revealed that the enhancement of resistance depends on OsCERK1 (Figure S4). As OsCERK1 is involved in the recognition of fungi and bacteria [16], receptor complexes containing OsCERK1 could be fully responsible for the activation of BSR1-mediated resistance. Meanwhile, many other RLKs homologous to OsCERK1 are involved in PTI [1,2]. These RLKs may also be involved in resistance enhancement, which requires further investigation.

In conclusion, our data demonstrated that the MAMP-recognition system (at least, OsCERK1) is mechanistically involved in broad-spectrum disease resistance. Given the limited knowledge of broad-spectrum resistance mechanisms, our study provides a new insight into this type of resistance.

## 4. Materials and Methods

### 4.1. Plant and Microbial Materials and Inoculation

*Oryza sativa* L. ‘Nipponbare’ was used as the WT line. *Pyricularia oryzae* isolate Kyu89-246 (MAFF101506, race 003.0) was used as a compatible rice blast fungus strain. Fungal culture and spore inoculation were performed as previously described [7,12]. Briefly, one milliliter of a *P. oryzae* conidial suspension (1.0 × 10^5^ mL^−1^) was sprayed onto each plant at the 3.5–4.0 leaf stage. The number of compatible lesions on the 4th leaf blade was counted on day 6 after inoculation.

### 4.2. Plasmid Construction and Transformation

The CRISPR-P online tool (http://cbi.hzau.edu.cn/crispr/) [20] was used to design CRISPR/Cas9 system-mediated cleavage sequences. Two independent sequences on *OsCERK1* exon 1 were selected: 5′-CCTTCTACGTGACGCCGAACCAG-3′ (target site #4) and 5′-CCGGGTGCGACCTCGCGCTGGCT-3′ (target site #10) were located at 120–142 bp and 95–117 bp from the first nucleotide of the start codon, respectively. To construct the modified binary vectors, a DNA fragment containing the maize *Ubiquitin* promotor–*BSR1* cDNA–*NOS* terminator was PCR-amplified from the pRiceFOX:*BSR1* vector [6]. The fragment was inserted into the *Asc*I site of the CRISPR/Cas9 vector (pZH_MMCas9), [18] resulting in a T-DNA containing a *Cas9*-, *gRNA*-, and *BSR1*-constitutive expression cassette. The targeting region in *gRNA* was replaced with the sequence of sites #4 or #10. These vectors and pRiceFOX:*BSR1* were used for Agrobacterium (*Rhizobium radiobacter*)-mediated rapid transformation, as previously reported [21]. *OsCERK1* in genome DNA of T0 plant was sequenced using a primer 5′-AGCTTCCACCTCCCTCCTAGTC-3′ (OsCERK1-F primer) as previously described [11]. Western blot analysis using anti-BSR1 antibody were performed as previously described [12,13]. For sequencing the *OsCERK1* transcript, total RNA was extracted from WT, *BSR1*ox6 and the two *OsCERK1*ko:*BSR1*ox plants, and reverse-transcribed using ReverTra Ace qPCR RT Master Mix with gDNA Remover (Toyobo, Osaka, Japan). DNA fragments containing the CRISPR/Cas9 system target site and neighboring region were amplified by PCR using OsCERK1-F primer and a primer 5′-GGTGTCCGGGATGTTGTT-3′, and sequenced using a primer 5′-GCGGTGGTGAGGTTGTTGTAG-3′.

### 4.3. Assay of MAMP-Responsivity

Leaf strips were prepared from the second leaves of two-month old plants. Two fragments of each leaf blade (8-mm length and 6-mm width) were slit using bundled razor blades [22] and placed in sterile water in a 12-well plate. Treatment with 100 nM chitin hexamer (*N*-acetylchitohexaose, GN6) and 10 µg mL^−1^ peptidoglycan from *Bacillus subtilis* (Sigma-Aldrich, St. Louis, MO, USA), and determination of the H_2_O_2_ concentration using luminol-dependent chemiluminescence assay, were conducted as previously described [12]. Transcript levels of defense-related genes at 5 h after treatment were assessed by RT-qPCR, using the comparative C_T_ (2^−ΔΔCt^) method [23]. *PBZ1* and *KSL4* were used as chitin-induced marker genes [11]. Rice *Ubiquitin1* (*RUBQ1*; Os06g0681400) was used as an internal control [24].

## Figures and Tables

**Table 1:** Representative *oscerk1* mutations induced by transformation with an *OsCERK1*-knockout/*BSR1*-overexpression vector.

Target	Line	Cleavage Site Sequence	Frameshift
(Site #4)	WT	-GCTGGCTTCCTTCTACGTGACGCCGAACCAGAACGTCAC-	
	Transformant#4-2	-GCTGGCTTCCTTCTaACGTGACGCCGAACCAGAACGTCAC-	+1
		-GCTGGCTTCCTTCT-CGTGACGCCGAACCAGAACGTCAC-	−1
	Transformant#4-4	-GCTGGCTTCCTTCTaACGTGACGCCGAACCAGAACGTCAC-	+1
		-GCTGGCTTCCTTCTaACGTGACGCCGAACCAGAACGTCAC-	+1
(Site #10)	WT	-GTGCAGCGCCGGGTGCGACCTCGCGCTGGCTTCCTTCTA-	
	Transformant#10-2	-GTGCAGCGCCGGGTtGCGACCTCGCGCTGGCTTCCTTCTA-	+1
		-GTGCAGCGCCGGGTtGCGACCTCGCGCTGGCTTCCTTCTA-	+1
	Transformant#10-6	-GTGCAGCGCCGGGT--GACCTCGCGCTGGCTTCCTTCTA-	−2
		-GTGCAGCGCCGGGTaGCGACCTCGCGCTGGCTTCCTTCTA-	+1

Underlined strings, CRISPR/Cas9-mediated cleavage sequences; lower-case letters and hyphens, insertion–deletion mutations found in the T0 plants.

## Supplementary Materials

Supplementary materials can be found at https://www.mdpi.com/1422-0067/21/15/5397/s1. Figure S1: Schematic structure of the translation products of wild-type *OsCERK1* and mutated *OsCERK1*; Figure S2: Sequences of *OsCERK1* transcript in WT, *OsCERK1*ko:*BSR1*ox lines, and *BSR1*ox6; Figure S3: Knockout of *OsCERK1* suppressed peptidoglycan-triggered H_2_O_2_ production in the *BSR1*-overexpressing background; Figure S4: The enhancement of disease resistance by *BSR1* overexpression depends on OsCERK1.
