# Supplementary material for: Overexpressed BSR1-Mediated Enhancement of Disease Resistance Depends on the MAMP-Recognition System
Source: Int J Mol Sci. 2020 Jul 29;21(15):5397. doi: 10.3390/ijms21155397 (PMC7432911; doi:10.3390/ijms21155397)
Supplement: Supplementary file 1 [file ijms-21-05397-s001.pdf]

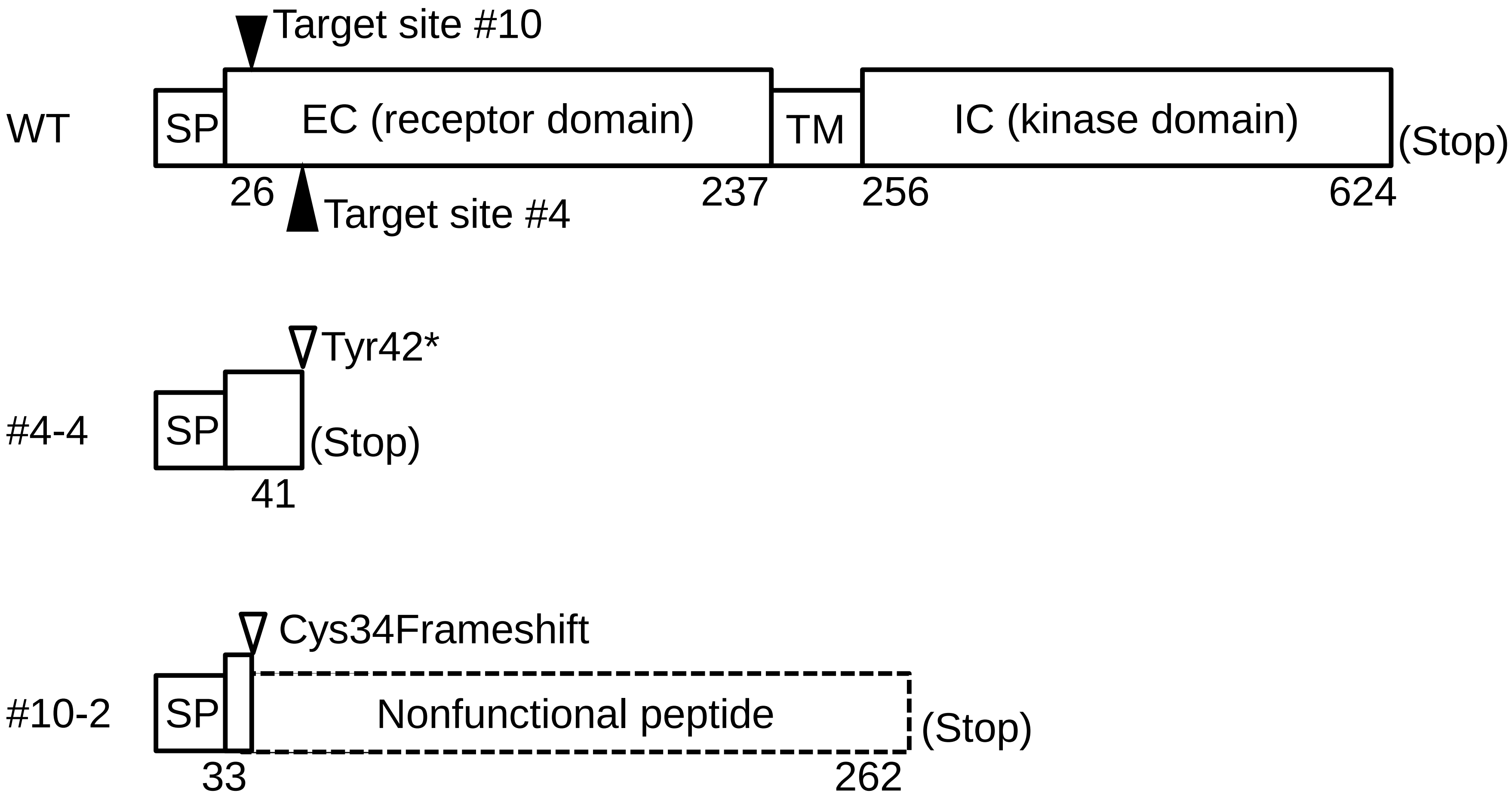

Figure S1. Schematic structure of the translation products of wild-type *OsCERK1* and mutated *OsCERK1*. Numerals under boxes indicate the number of amino acid residues. Black arrowheads, positions corresponding to CRISPR/Cas9 system-targeted sites; white arrowheads, insertion mutation-mediated amino acid substitution; SP, signal peptide; EC, extracellular domain; TM, transmembrane domain; IC, intracellular domain; Stop, stop codon.

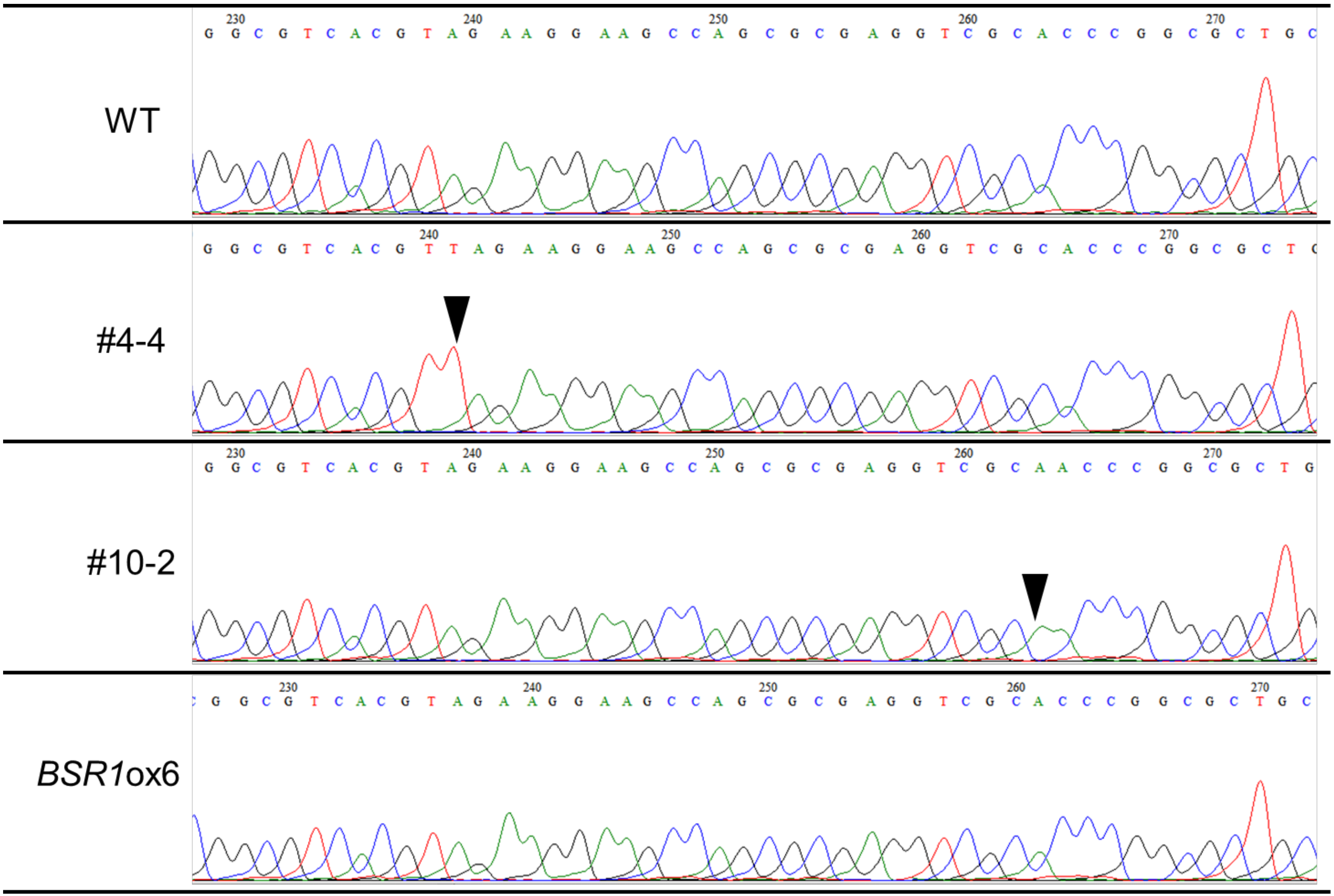

Figure S2. Sequences of *OsCERK1* transcript in WT, *OsCERK1*ko:*BSR1*ox lines, and *BSR1*ox6. Complementary strand was sequenced after reverse-transcription reaction. Black arrowheads, CRISPR/Cas9 system-mediated insertion mutations.

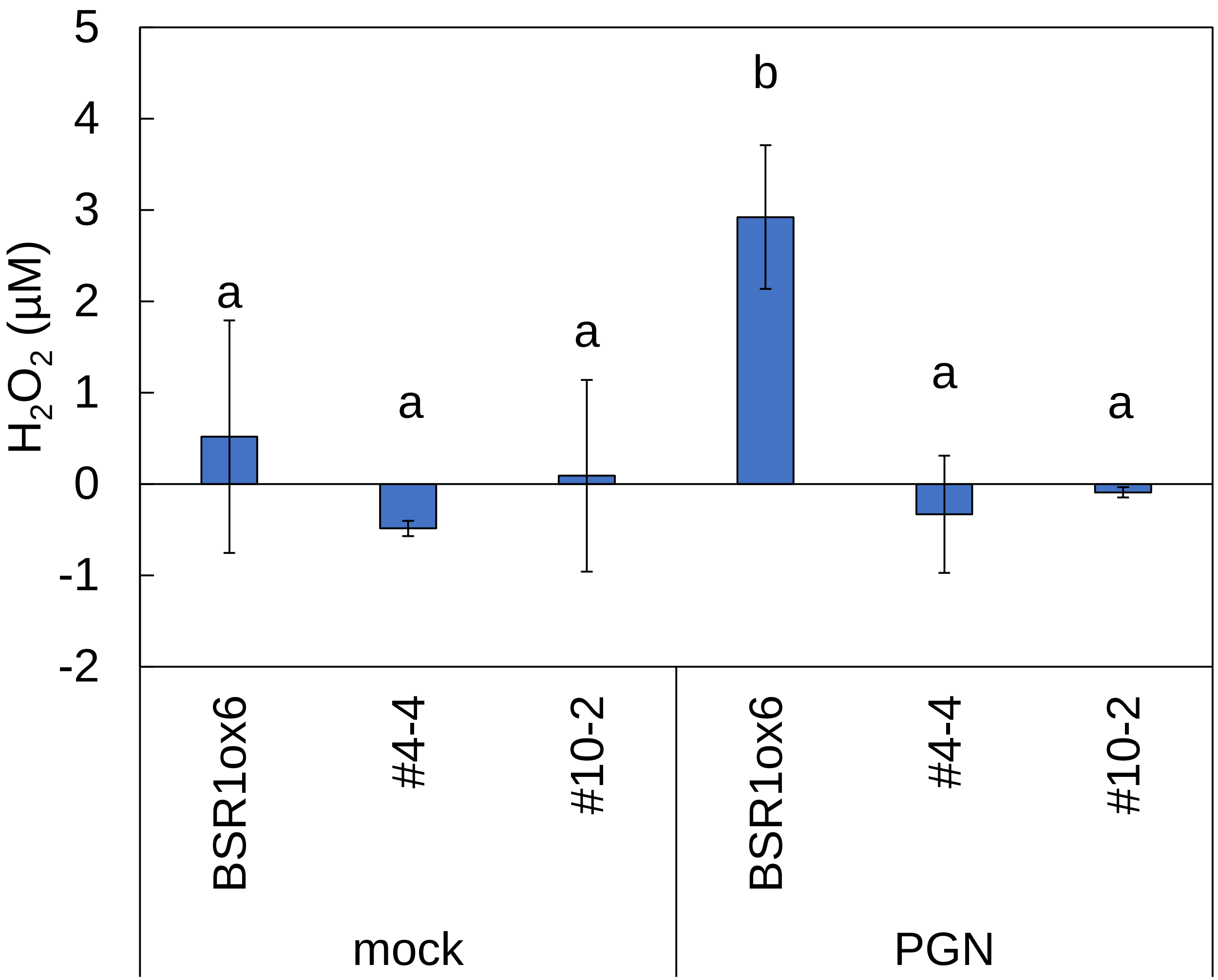

Figure S3. Knockout of *OsCERK1* suppressed peptidoglycan-triggered H_2O_2 production in the *BSR1*-overexpressing background. H_2O_2 concentrations were measured by luminol-dependent chemiluminescence assay at 120 min after treatment. Values are presented as the means ± standard deviations of three biological replicates. Experiments were conducted twice, with similar results. Different letters indicate significant differences (Tukey's test; $p < 0.05$). Mock, treatment with water; PGN, treatment with peptidoglycan.

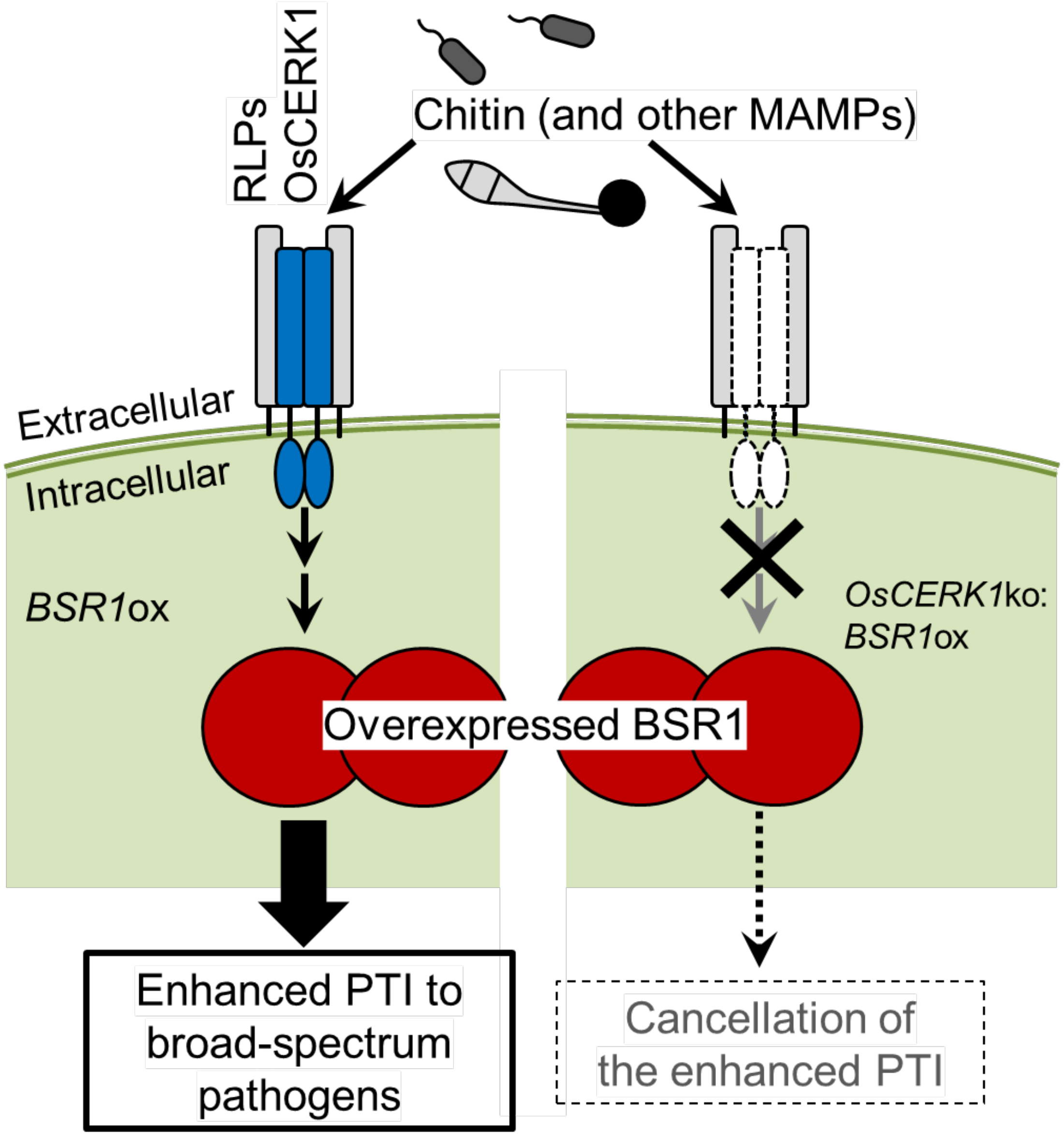

Figure S4. The enhancement of disease resistance by *BSR1* overexpression depends on OsCERK1. RLPs, receptor-like proteins; MAMPs, microbe-associated molecular patterns; PTI, pattern-triggered immunity.
